# Orbital angular momentum multiplexing holography based on multiple polarization channel metasurface

**DOI:** 10.1515/nanoph-2023-0550

**Published:** 2023-11-09

**Authors:** Yue Wang, Zhenyu Yao, Zijian Cui, Guangcheng Sun, Dachi Zhang

**Affiliations:** Key Laboratory of Ultrafast Photoelectric Technology and Terahertz Science in Shaanxi, Xi’an University of Technology, Xi’an, 710048, China

**Keywords:** optical nested encryption, multiplexing holography, OAM multiplexing, metasurfaces, terahertz

## Abstract

As a high-degree-of-freedom approach to manipulate the electromagnetic wave, metasurfaces are widely used in high-capacity information technology. Extensive investigations have explored multiplexing techniques using polarization, incident angle, wavelength, and infinite-dimensional multiplexing through Orbital Angular Momentum (OAM). However, due to the limited spatial resolution and array size of the metasurface, the number of multiplexing channels that can be actually realized is limited. Therefore, research on the combination of OAM multiplexing and polarization degrees of freedom is of great significance. Here, we propose and experimentally demonstrate a metasurface holography multiplexing scheme based on multiple polarization channels combined with OAM. Taking advantage of the orthogonal independence of spin angular momentum and orbital angular momentum, multiple OAM multiplexing holograms are constructed in multiple different spin-polarization channels. Utilizing the well-established compatibility between OAM multiplexing and polarization multiplexing, we successfully integrated two multiplane holograms and 15 OAM multiplexing holograms on a single metasurface. Subsequently, we introduced an optical nested encryption framework designed for parallel communication. This work facilitates high-capacity and high-security holography by employing multiplexing metasurfaces, thereby providing innovative design concepts for optical communication, information encryption, and related domains.

## 1 Introduction

For recording and reproducing wavefronts, holography is widely used across the electromagnetic spectrum from microwaves to visible light [1–3]. Due to its flexibility in implementation and control capabilities, holography has tremendous potential applications in areas such as optical tweezers [4], three-dimensional display [5], all-optical storage [6], all-optical computing [7, 8], beam shaping [9], optical cloak [10], optical encryption [11–13], and virtual reality/augmented reality display [14, 15]. The research hotspots of holographic applications are developing towards multifunctional, reconfigurable, miniaturized, and integrated directions. Although there are several feasible techniques for implementing holography, including spatial light modulators and digital micromirror devices, its applications are still limited by its large spatial volume and lower working efficiency. Generally, the wavefront recorded in the hologram contains information of amplitude, phase, and polarization of the electromagnetic fields. Therefore, the realization of the multiplexing of the hologram is inseparable from the multiplexing of the multi-dimensional control of the light field. Common control strategies use degree of freedom (DoFs) of light such as wavelength, polarization, incident angle, optical structure, and level of irradiance as control dimensions [16]. These multi-dimensional control strategies manipulate the incident light [17, 18], modulator [19, 20], and outgoing light [21, 22] to achieve hologram multiplexing.

Metasurfaces consisting of sub-wavelength structures have several advantages such as ultra-thin, high efficiency, complete control of electromagnetic waves. Based on its powerful capability to manipulate the degree of freedom (DoFs) of waves such as amplitude and phase, the research on metasurfaces for holographic multiplexing has attracted great attention. However, single-function metasurface holography can only generate one holographic image, which limits the application. Therefore, it is necessary to introduce multiplexing technology into metasurface holography. In order to realize multiplexing holography, multi-dimensional multiplexing approaches including wavelength [23–25], incident angle [26], polarization state [27–29], and time [30] have all been demonstrated on the metasurfaces platform. However, for 2D planar metasurfaces that can be represented by a Jones matrix with different off-diagonal elements and same diagonal element, there is a theoretical upper limit on the number of DoFs, which greatly restricts the increase in multiplexing channels [31]. Furthermore, the fundamental limitation of polarization multiplexing capacity in metasurfaces, rooting from the dimensional constraints of the Jones matrix, has been overcome by introduction of engineered noise for suppression crosstalk [32]. Recently, the concept of multiplexed holography based on the orbital angular momentum (OAM) of photons in the metasurface is proposed [33–36]. Because of the infinite number of orthogonal helical modes of OAM beam, OAM-based holography can realize the multiplexed holography of infinite orthogonal channels in theory [37]. However, the number of OAM multiplexed holography channels is limited by the spatial resolution and arrays size of the metasurface [38]. A comprehensive multiplexing approach is needed to enable metasurfaces to generate a greater number of OAM multiplexed holograms channels, which is of great significance for the application of OAM multiplexed holograms.

In this work, we propose an OAM multiplexing holography technique based on an all-dielectric terahertz metasurface that achieve complex-amplitude modulation in multiple polarization channels. The proposed metasurface has the capability to independently control the amplitude and phase of the transmitted components of circularly polarized waves in the co-polarization and cross-polarization channels, which can freely achieve different types of OAM multiplexing holography in different channels. By incorporating cylindrical and elliptical cylindrical structures into a super-pixel, we achieve simultaneous and independent manipulate of the complex amplitudes in three polarization channels: co-polarization channel, left circular polarization–right circular polarization (LCP–RCP), and right circular polarization–left circular polarization (RCP–LCP). To demonstrate the compatibility of our proposed metasurface platform with OAM holography, we investigate three specific scenarios: lens-less multi-plane holography with an OAM helical phase feature of *l* = 0; OAM multiplexed lens-less holography on multi-plane; and OAM multiplexed Fourier holograms. These scenarios correspond to the three polarization channels of the proposed metasurface. The measured results are in good agreement with the simulated results. Our proposed metasurface design provides rich and flexible independent channels based on spin angular momentum (SAM) and OAM, which provides a platform for realizing high-capacity and high-security optical nested encryption. We employ a nested encryption technique to secure optical information. This involves utilizing both public and private keys to transform the data into a multi-channel OAM multiplexed hologram, which is then integrated onto a singular metasurface. By combining the double-key encryption and multi-channel OAM multiplexing method, our strategy supports the construction of multiple high-capacity encryption channels, which enables simultaneous communication among multiple users, while ensuring the confidentiality of the encrypted information.

**Figure 1:**
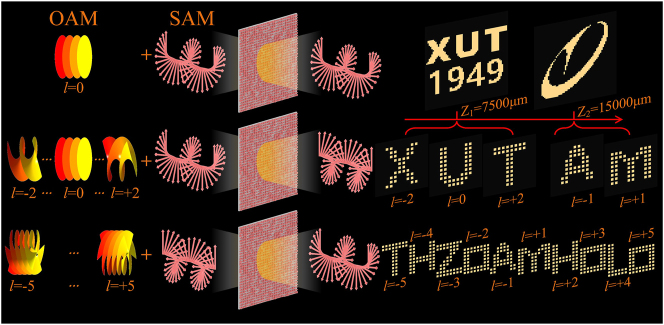
Schematic diagram of OAM multiplexing holography based on multiple polarization channel complex-amplitude modulation metasurface. This metasurface has the capability to manipulate the complex-amplitude of both the transmitted co-polarization component and the cross-polarization component of an incident circularly polarized wave. Two independent holograms are presented at different *Z* planes for the corresponding co-polarized transmission component when an arbitrary circularly polarized wave is incident. Five distinct OAM holograms are exhibited by the RCP component of the transmitted wave for LCP incidence at different *Z* planes. A 10-order OAM multiplexed hologram is displayed by the LCP component of the transmitted wave for RCP incidence.

Figure 1 shows the conceptual schematic diagram of the OAM multiplexing holography based on multiple polarization channel complex-amplitude modulation metasurface. By controlling the polarization states of the incident wave and the outgoing wave, three different types of OAM multiplexing holograms can be realized. Specifically, for the co-polarized transmitted wave of the circularly polarized incident wave with an arbitrary helical mode index of zero, the holographic image can be reconstructed at different depths along the wave path; Secondly, for the RCP transmitted wave of the LCP incident wave of the OAM mode with the helical mode index (l), when l = +2, ∼ −2, OAM multiplexing holographic images representing letters ‘X’, ‘U’, ‘T’, ‘A’, ‘M’ can be reconstructed, respectively, at different depths along the wave path; Finally, for the OAM mode RCP incident wave whose helical mode index (*l*) is −5∼+5, the outgoing LCP wave can, respectively, reconstruct 10 different holographic images, corresponding to the string ‘THZOAMHOLO’ (representing ‘Terahertz OAM Holography’).

## 2 Results and discussions

**Figure 2:**
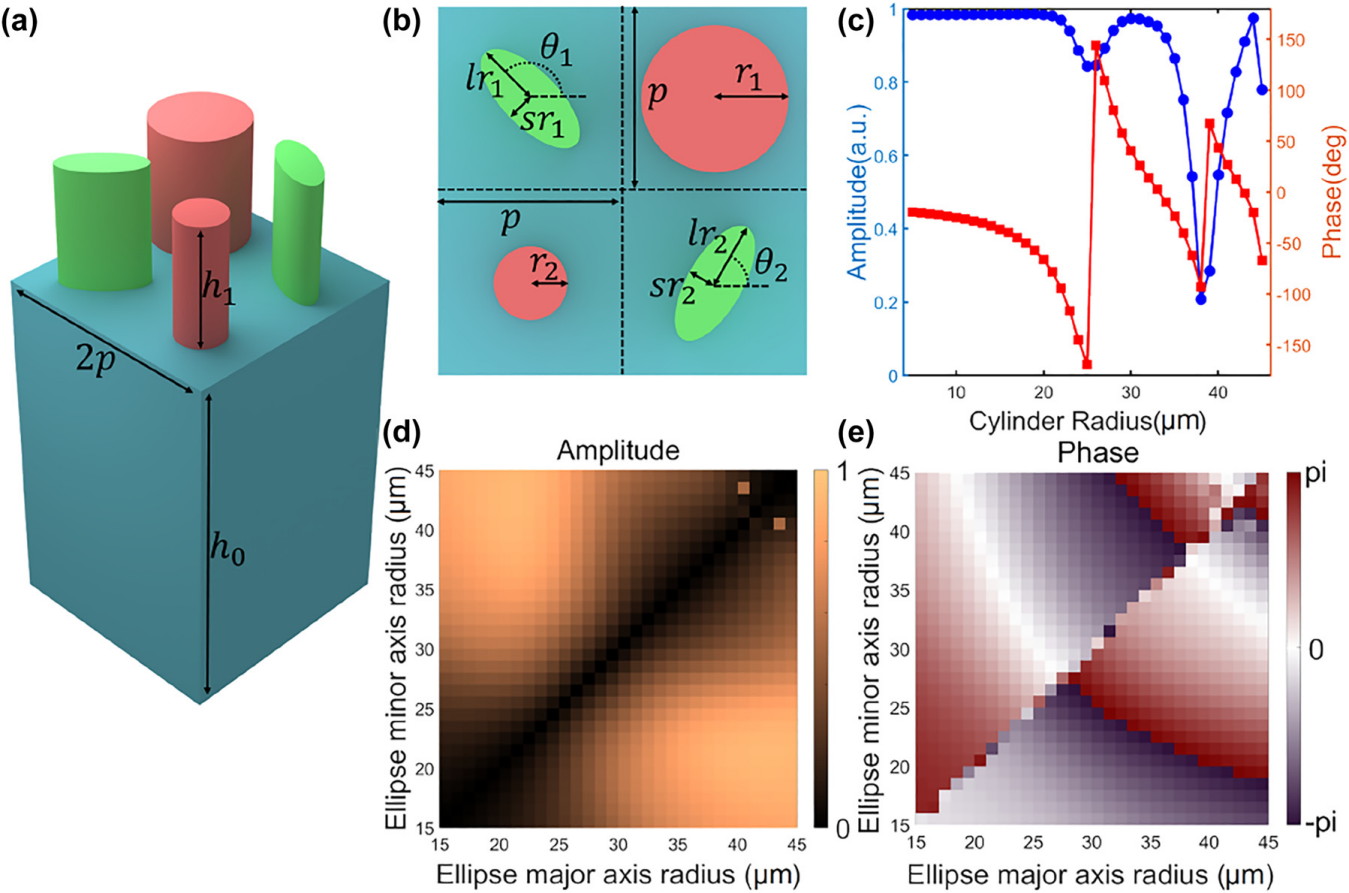
The design of the super-pixel in the proposed metasurface. (a) The super-pixel comprises cylindrical co-polarized wave control structures (red) and elliptical cylindrical cross-polarized wave control structures (green). (b) Schematic diagram illustrating the structural parameters of the super-pixel. (c) The amplitude and phase of the co-polarized transmission wave for cylindrical structures of different diameters obtained through simulation. (d) Amplitude and (e) phase of cross-polarized transmitted waves for elliptical cylindrical structures of varying geometric sizes are shown.

In order to simultaneously manipulate the co-polarization and cross-polarization transmission channels of the metasurface, we propose a super-pixel structure as shown in Figure 2(a), which consists of an isotropic cylindrical structure and an anisotropic elliptical cylindrical structure. The period of the super-pixel is 2*p* = 200 μm, the cylinder and the ellipse are made of silicon with a dielectric constant of 11.9, the height of the silicon structure is *h*_1_ = 100 μm, and the thickness of the substrate material is *h*_0_ = 500 μm quartz with a refractive index of 1.45. For the circular polarization incident wave, the isotropic cylindrical structure will not generate cross-polarized transmitted waves, and other anisotropic elliptical cylindrical structures that can be regarded as half-wave plates will hardly generate co-polarized transmitted waves. Therefore, the co-polarized transmitted wave is completely regulated by the cylindrical structure, and the cross-polarized transmitted wave is completely regulated by the elliptic cylindrical structure. By designing the cylinder and the ellipse cylinder together in the super-pixel, the independent regulation of the co-polarization and cross-polarization transmission channels is realized.

For the proposed metasurface, its Jones matrix under the circular polarization basis vector can be expressed as:

(1) $$\begin{array}{ll} J & =\left[\begin{array}{cc} A_{\text{co}}{\text{e}}^{\text{i}\varphi_{\text{co}}} & A_{lr}{\text{e}}^{\text{i}\varphi_{lr}}\\ A_{rl}{\text{e}}^{\text{i}\varphi_{rl}} & A_{\text{co}}{\text{e}}^{\text{i}\varphi_{co}} \end{array}\right]\\ & =\left[\begin{array}{cc} A_{\text{co}}{\text{e}}^{\text{i}\varphi_{\text{co}}} & 0\\ 0 & A_{\text{co}}{\text{e}}^{\text{i}\varphi_{\text{co}}} \end{array}\right]+\left[\begin{array}{cc} 0 & A_{lr}{\text{e}}^{\text{i}\varphi_{lr}}\\ A_{rl}{\text{e}}^{\text{i}\varphi_{rl}} & 0 \end{array}\right]\\ & =J_{\text{co}}+J_{\text{cr}} \end{array}$$

where ***J***_co_ is the Jones matrix of the co-polarization channel, and ***J***_cr_ is the Jones matrix of the cross-polarization channel. In order to further realize the complex amplitude regulation of the three polarization multiplexing channels, we design two cylindrical and two elliptical cylindrical structures with different parameters in a super-pixel. The phase modulation is transformed into complex amplitude modulation through the interference phenomenon between the two transmitted waves. Mathematically, a complex amplitude with amplitude *A*_0_ and phase *φ*_0_ can be decomposed into the sum of two complex amplitudes with equal amplitude and different phase:

(2) $$\begin{array}{ll} A_{0}{\text{e}}^{\text{i}\varphi_{0}} & =\frac{A_{0}}{2\cos\varphi_{1}}\left({\text{e}}^{\text{i}\varphi_{1}}+e^{-i\varphi_{1}}\right){\text{e}}^{\text{i}\varphi_{0}}\\ & =\frac{A_{0}}{2\cos\varphi_{1}}{\text{e}}^{\text{i}\left(\varphi_{0}+\varphi_{1}\right)}+\frac{A_{0}}{2\cos\varphi_{1}}{\text{e}}^{\text{i}\left(\varphi_{0}-\varphi_{1}\right)} \end{array}$$

Therefore, the Jones matrix ***J***_co_, ***J***_cr_ can be further decomposed:

(3) $$\begin{array}{ll} J_{\text{co}} & =\left[\begin{array}{cc} A_{\text{co}}{\text{e}}^{\text{i}\varphi_{\text{co}}} & 0\\ 0 & A_{\text{co}}{\text{e}}^{\text{i}\varphi_{\text{co}}} \end{array}\right]\\ & =\left[\begin{array}{cc} {\text{e}}^{\text{i}\left(\varphi_{\text{co}}+\varphi_{1}\right)} & 0\\ 0 & {\text{e}}^{\text{i}\left(\varphi_{\text{co}}+\varphi_{1}\right)} \end{array}\right]+\left[\begin{array}{cc} {\text{e}}^{\text{i}\left(\varphi_{\text{co}}-\varphi_{1}\right)} & 0\\ 0 & {\text{e}}^{\text{i}\left(\varphi_{\text{co}}-\varphi_{1}\right)} \end{array}\right]\\ & =\left[\begin{array}{cc} {\text{e}}^{\text{i}\varphi_{\text{co}1}} & 0\\ 0 & {\text{e}}^{\text{i}\varphi_{\text{co}1}} \end{array}\right]+\left[\begin{array}{cc} {\text{e}}^{\text{i}\varphi_{\text{co}2}} & 0\\ 0 & {\text{e}}^{\text{i}\varphi_{\text{co}2}} \end{array}\right]\\ & =J_{\text{co}1}+J_{\text{co}2} \end{array}$$

(4) $$\begin{array}{ll} J_{\text{cr}} & =\left[\begin{array}{cc} 0 & A_{lr}{\text{e}}^{\text{i}\varphi_{lr}}\\ A_{rl}{\text{e}}^{\text{i}\varphi_{rl}} & 0 \end{array}\right]\\ & =\left[\begin{array}{cc} 0 & {\text{e}}^{\text{i}\left(\varphi_{lr}+\varphi_{2}\right)}\\ {\text{e}}^{\text{i}\left(\varphi_{rl}+\varphi_{3}\right)} & 0 \end{array}\right]+\left[\begin{array}{cc} 0 & {\text{e}}^{\text{i}\left(\varphi_{lr}-\varphi_{2}\right)}\\ {\text{e}}^{\text{i}\left(\varphi_{rl}-\varphi_{3}\right)} & 0 \end{array}\right] \end{array}$$

where cos(*φ*_1_) = *A*_co_/2, cos(*φ*_2_) = *A*_*lr*_/2, cos(*φ*_3_) = *A*_*rl*_/2. For the Jones matrix under the circular polarization basis vector, the role of the rotation matrix *M*(*φ*) presents as opposite phase modulation in the cross-polarized channels [39].

(5) $$\begin{array}{ll} M\left(\varphi \right) & =\left[\begin{array}{cc} {\text{e}}^{\text{i}\varphi } & 0\\ 0 & e^{-i\varphi } \end{array}\right];\;M\left(\varphi \right)JM{\left(\varphi \right)}^{-1}\\ & =\left[\begin{array}{cc} E_{ll} & E_{lr}e^{i2\varphi }\\ E_{rl}e^{-i2\varphi } & E_{rr} \end{array}\right] \end{array}$$

***J***_cr_ can be further expressed as:

(6) $$\begin{array}{ll} J_{\text{cr}} & =M\left(\theta_{1}\right)\left[\begin{array}{cc} 0 & {\text{e}}^{\text{i}\varphi_{\text{cr}1}}\\ {\text{e}}^{\text{i}\varphi_{\text{cr}1}} & 0 \end{array}\right]M{\left(\theta_{1}\right)}^{-1}+M\left(\theta_{2}\right)\\ & \;\times \left[\begin{array}{cc} 0 & {\text{e}}^{\text{i}\varphi_{\text{cr}2}}\\ {\text{e}}^{\text{i}\varphi_{\text{cr}2}} & 0 \end{array}\right]M{\left(\theta_{2}\right)}^{-1}=J_{\text{cr}1}+J_{\text{cr}2} \end{array}$$

where *φ*_cr1_ = (*φ*_*lr*_ + *φ*_2_ + *φ*_*rl*_ + *φ*_3_)/2, *φ*_cr2_ = (*φ*_*lr*_ − *φ*_2_+*φ*_*rl*_ − *φ*_3_)/2, *θ*_1_ = (*φ*_*lr*_ + *φ*_2_ − *φ*_*rl*_ − *φ*_3_)/4, *θ*_2_ = (*φ*_*lr*_ − *φ*_2_ − *φ*_*rl*_ + *φ*_3_)/4. The Jones matrix of the proposed polarization multiplexing metasurface can be expressed as ***J*** = ***J***_co1_ + ***J***_co2_ + ***J***_cr1_ + ***J***_cr2_, among them, ***J***_co1_ and ***J***_co2_ can be realized by isotropic cylinders, ***J***_cr1_ and ***J***_cr2_ can be realized by the half-wave plate elliptical cylinders which are, respectively, rotated by specific angles *θ*_1_ and *θ*_2_. As shown in Figure 2(b), the radius of the two cylindrical structures are *r*_1_ and *r*_2_, respectively, and the lengths of the major and minor semi-axes of the two elliptical cylindrical structures are *lr*_1_, *sr*_1_, *lr*_2,_ *sr*_2_, respectively. The rotation angles of the elliptical cylinder are *θ*_1_ and *θ*_2_, respectively. At 1.3 THz, the amplitude and phase of the transmitted wave in co-polarization channel of the cylinders with different radius are shown in Figure 2(c), and the amplitude and phase of the transmitted wave in cross-polarization channel of elliptical cylinders with different geometric sizes are shown in Figure 2(d) and (e), respectively.

**Figure 3:**
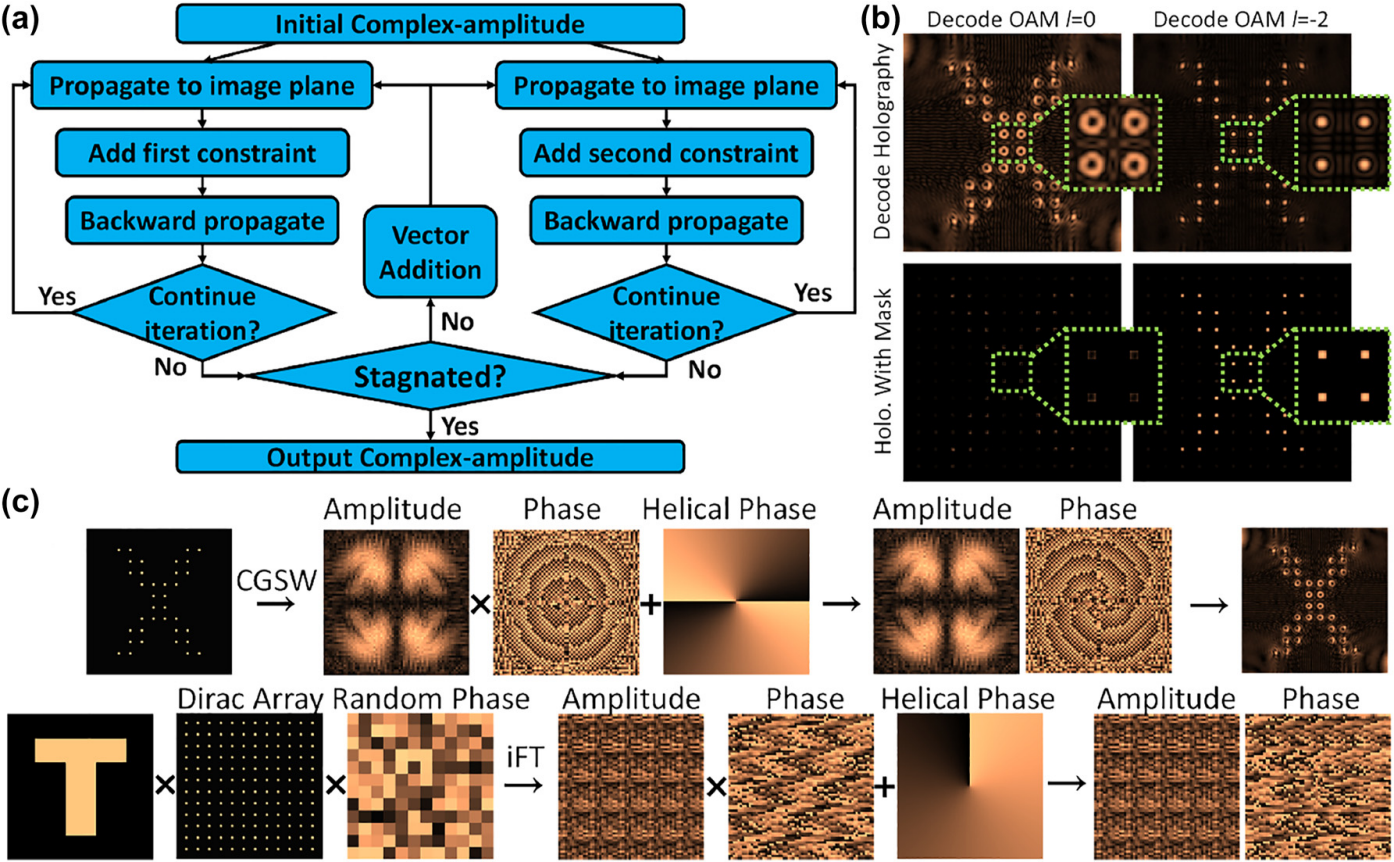
Design principles of OAM-based holograms. (a) Schematic diagram of the spatial multiplexing complex-amplitude weighted-GS(CGSW) algorithm. (b) Schematic diagram of Selective OAM holography, where the OAM selective mask samples the holographic image to ensure that the OAM hologram generated in (c) is exclusively displayed in the specified decoding OAM channel. (c) The construction process of a lens-less OAM hologram and an OAM hologram based on Fourier transform.

Based on the proposed complex-amplitude-modulating metasurface, we used the complex-amplitude weighted-Gerchberg–Saxton (CGSW) algorithm to obtain lens-less holograms in the form of complex amplitude. The algorithm enables calculation of the field distribution in propagation using the Rayleigh–Sommerfeld diffraction formula and improves the imaging quality by enlarging the size of the imaging plane in iterations [40, 41]. In order to achieve the complex amplitude modulation that can independently control the field on the two planes, we use the independent multiple-iteration CGSW algorithm shown in Figure 3(a). This algorithm generates new shared complex amplitude for subsequent independent iterations by vector adding the complex amplitudes obtained by multiple independent iterations between the two imaging planes and the metasurface. The construction process of OAM holography is shown in Figure 3(c). For the OAM hologram without a lens, the amplitude and phase of the hologram are first obtained by the CGSW algorithm, and then the helical phase of the OAM beam is superimposed to obtain the OAM hologram. It can be seen that the reconstructed holographic image at this time is composed of a series of pixels with donut intensity distribution. Similar to the lens-less OAM hologram, the Fourier OAM hologram is obtained by sampling the image using a 2D Dirac array with random phases and inverse Fourier transforming of the sampled image. When the topological charge number of the incident decoding wave is opposite to the helical phase feature value *l* added during the construction of the OAM hologram, the pixels with the donut intensity distribution in the reconstructed hologram will degenerate into pixels with a Gaussian mode. When the same 2D Dirac array is used to process the reconstructed holographic image, only the pixels with the Gaussian mode formed by specific incident topological charge number can appear, as shown in Figure 3(b), thereby OAM selective holography is realized.

**Figure 4:**
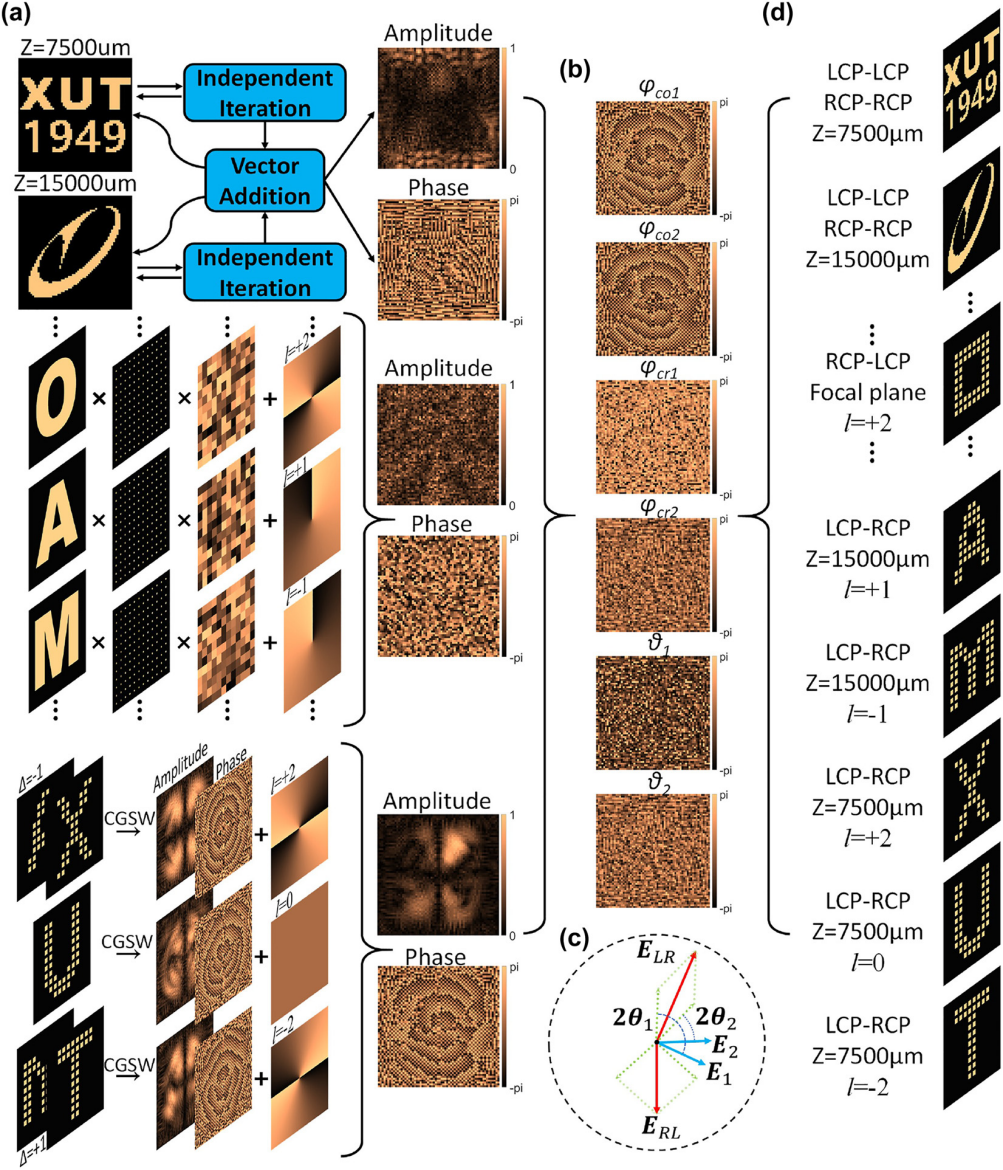
Schematic diagram of the implementation of OAM multiplexed holography in a multiple polarization channel complex-amplitude modulation metasurface. (a) Encoding holograms obtained through different methods into complex-amplitude wavefronts that the metasurface needs to achieve across three polarization channels. (b) Illustration of the four propagation phases and two rotation angles of four resonant structures within a super-pixel, acquired through the encoding of complex amplitudes across three polarization channels. (c) Schematic diagram depicting spin decoupling for complex-amplitude modulation in cross-polarized channels. (d) Demonstration of OAM multiplexing holography based on the designed multiple-polarization-channel complex-amplitude modulated metasurface.

Due to the mutual orthogonality between SAM and OAM of photons, the polarization multiplexing scheme based on SAM can be combined with OAM multiplexing to realize polarization multiplexing OAM holography [42]. Different polarization multiplexing channels depend on incident waves and detection waves with different SAMs, while OAM multiplexing depends on the cooperation of incident OAM and sampling arrays. Therefore, OAM-based multiplexing holography can be freely constructed in different polarization multiplexing channels at a working frequency of 1.3 THz. As shown in Figure 4, we constructed a multiplane multiplexed hologram, a lens-less OAM multiplexed hologram, and an OAM multiplexed Fourier hologram in one co-polarized channel and two cross-polarized channels, respectively. As shown in Figure 4(a), the OAM multiplexing holograms of each order in each polarization multiplexing channel are added separately to obtain the complex amplitude modulation that needs to be realized in the corresponding polarization channel. In the LCP-RCP channel, we construct holographic images corresponding to different OAM modes in two distinct z-planes through the previously proposed independent multiple-iteration CGSW algorithm. The complex amplitude modulations in these three polarization channels correspond to the three complex amplitude modulations in the Jones matrix ***J*** of metasurface in Eq. (1). Using Eqs. (1)–(6), we can decompose the ***J*** into *φ*_cr1_, *φ*_cr2_, *φ*_co1_, *φ*_co2_, *θ*_1_, and *θ*_2_, as shown in Figure 4(b). The intuitive representation of the relationship between complex amplitude modulation ***E***_*lr*_, ***E***_*rl*_ and *φ*_co1_, *φ*_co2_, *θ*_1_, *θ*_2_ in cross-polarized channels under ideal conditions is shown in Figure 4(c), where ***E***_1_ = exp(i*φ*_cr1_), ***E***_2_ = exp(i*φ*_cr2_). Under the corresponding polarization channel and OAM beam incidence, the proposed metasurface can achieve the corresponding reconstructed holographic image, as shown in Figure 4(d).

Unlike continuous complex amplitude modulation feasible in ideal conditions, metasurfaces in practice are limited to discrete complex amplitude modulation within a specific range. To minimize the comprehensive complex amplitude error across the metasurface, we choose suitable structural parameters from the cell library depicted in Figure 2. Utilizing Eq. (7), we sum the absolute differences between the actual complex amplitude modulation and the intended complex amplitude modulation in each channel of the metasurface. This summation serves to quantify the quality of reconstruction. The introduction of an extra phase difference between the complex amplitude modulations of distinct channels leads to a reduction in the overall complex amplitude modulation error. In our specific design, this error reduction amounts to 40 %.

(7) $$\min\left\{\sum \left[\text{abs}\left(A_{\text{real}}\cdot {\text{e}}^{\text{i}\cdot \varphi_{\text{real}}}-A_{\text{ideal}}\cdot {\text{e}}^{\text{i}\cdot \varphi_{\text{ideal}}}\right)\right]\right\}$$

**Figure 5:**
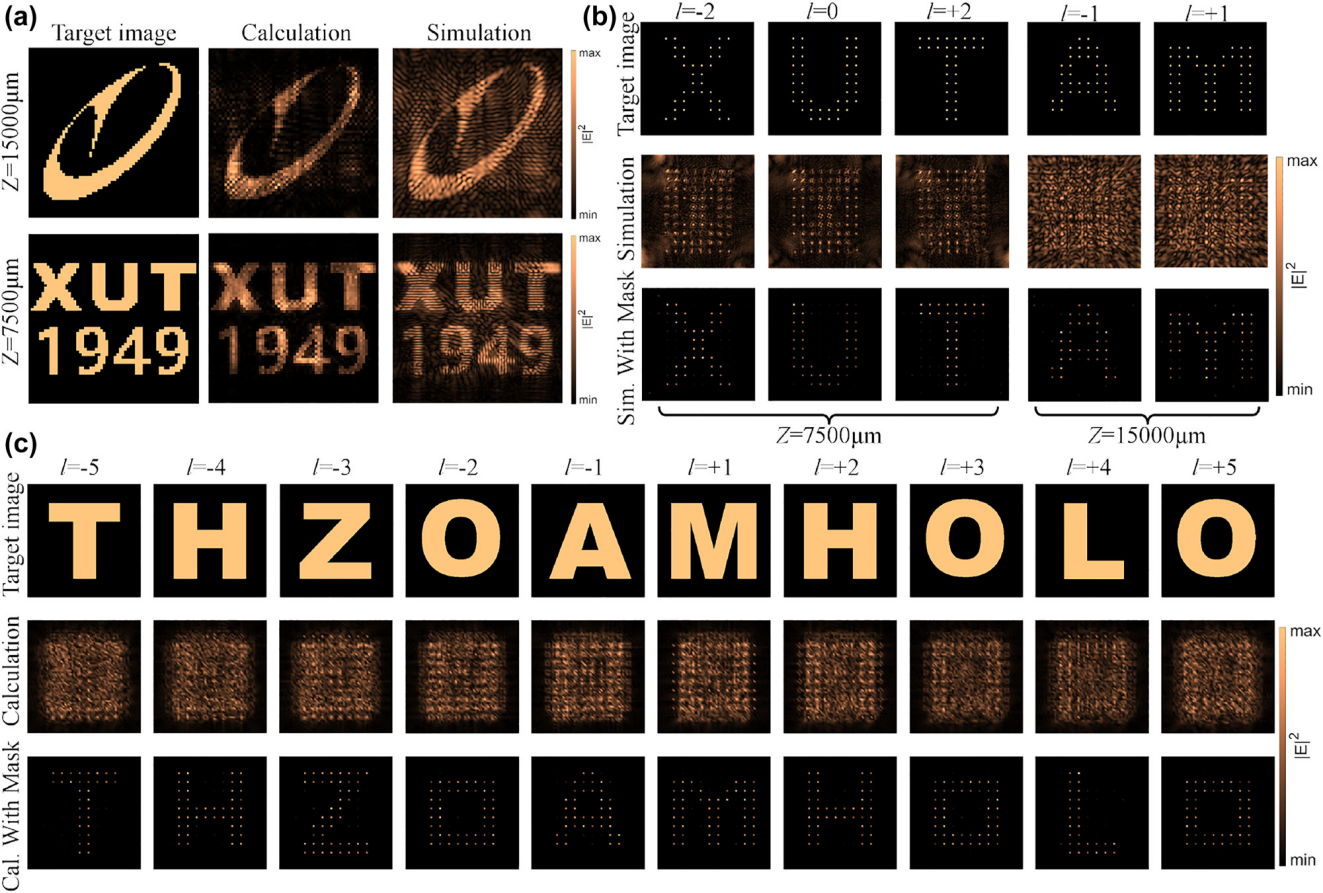
Characterization of polarization multiplexing OAM holograms. (a) Numerical and simulated reconstruction of holograms on two distinct *z*-planes in the co-polarized channel. (b) Numerical reconstruction of lens-less OAM holograms in the LCP-RCP polarization channel. (c) Numerical reconstruction of OAM holograms using the Fourier transform in the RCP-LCP polarization channel.

The final design of the multi-channel OAM multiplexing meta-hologram consists of 64 × 64 super-pixels, with an overall size of 12.8 mm × 12.8 mm. Figure 5(a) shows the lens-less multi-plane holographic image reconstructed in the co-polarization channel. The abbreviated characters representing Xi’an University of Technology are located at *Z* = 7500 μm, while the school badge pattern is positioned at *Z* = 15,000 μm. The simulation results are basically consistent with the theoretical calculation results. Figure 5(b) presents the reconstructed lens-less OAM holographic image in the LCP-RCP channel. To ensure that every pixel point sampled through the 2D Dirac mask can pass through the OAM selective mask when it has a zero topological charge, and to block pixel points that transform into donut patterns due to a non-zero topological charge, we chose the reconstructed holographic image corresponding to the 2D Dirac array as the most straightforward OAM selective mask meeting this requirement. The simulation demonstrates that the reconstructed holographic image faithfully restores the designed image after sampled by the OAM selective mask. The numerical reconstruction results of Fourier holography in the RCP-LCP channel are shown in Figure 5(c), and the designed tenth-order OAM-select holographic image can be well reconstructed.

**Figure 6:**
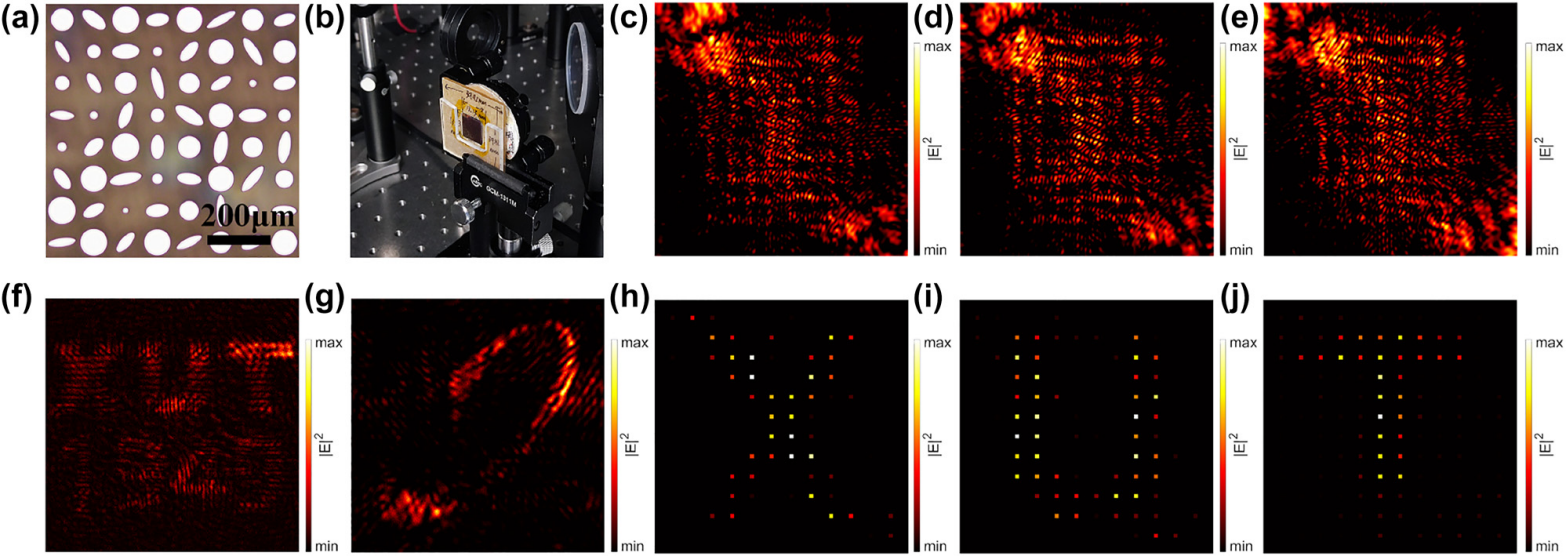
Experimental demonstration of polarization multiplexing OAM holograms. (a) Top views of the optical microscope image of the fabricated metasurface, with the scale bar to be 200 μm. (b) Sample holder with sample fixed in the test system. (f)–(g) Experimental reconstruction of holo-grams on two distinct *z*-planes, located 7500 μm and 15,000 μm away from the metasurface, respectively. (c)–(e) Experimental reconstruction of lens less OAM holograms with incident beams having different topological charges of +2, 0, −2. (h)–(j) Selective OAM holography obtained by sampling the holographic images in d-f with OAM selective mask.

The top view of the optical microscope image of the fabricated sample is shown in Figure 6(a). Here, holographic images reconstructed from metasurface were obtained by the THz digital holographic imaging system, and the sample fixed in the test system is shown in Figure 6(b). The measured result of the lens-less multi-plane holographic image is shown in Figure 6(f) and (g), respectively. The measured electric field distributions shown in Figure 6(f) is in good agreement with the simulation results at *z* = 7500 μm. The measurement results in Figure 6(g) only retain some features of the simulation results. In order to measure lens-less OAM Holography, we generate incident OAM beams through helical phase plates made of PTFE with topological charges of +2 and −2. Figure 6(c)–(e) shows the experimentally reconstructed lens-less OAM holographic images. The reconstructed holographic images obtained after sampling are shown in Figure 6(h)–(j). The three target images can be well reconstructed with the corresponding OAM beam incident. The experimental results have demonstrated the integration of the OAM multiplexing scheme with the multi-polarization channel metasurface, and the experimental results are in well agreement with the numerical results. Compared with the simulations, the experimental images depicted in Figure 5(f)–(j) manifest a discernible reduction in quality. We attribute this deterioration to an amalgamation of multiple reasons, encompassing crosstalk among distinct polarization channels, interference stemming from nanostructure coupling, non-uniform amplitude distribution of the incident Gaussian beam, misalignment between the incident beam and the metasurface, and imperfections in fabrication. Nevertheless, these challenges can be substantially alleviated, if not entirely eradicated, by augmenting the scale of the metasurface, enhancing its fabrication methodology, judiciously designing amplitude compensation, and optimizing the experimental optical setup.

**Figure 7:**
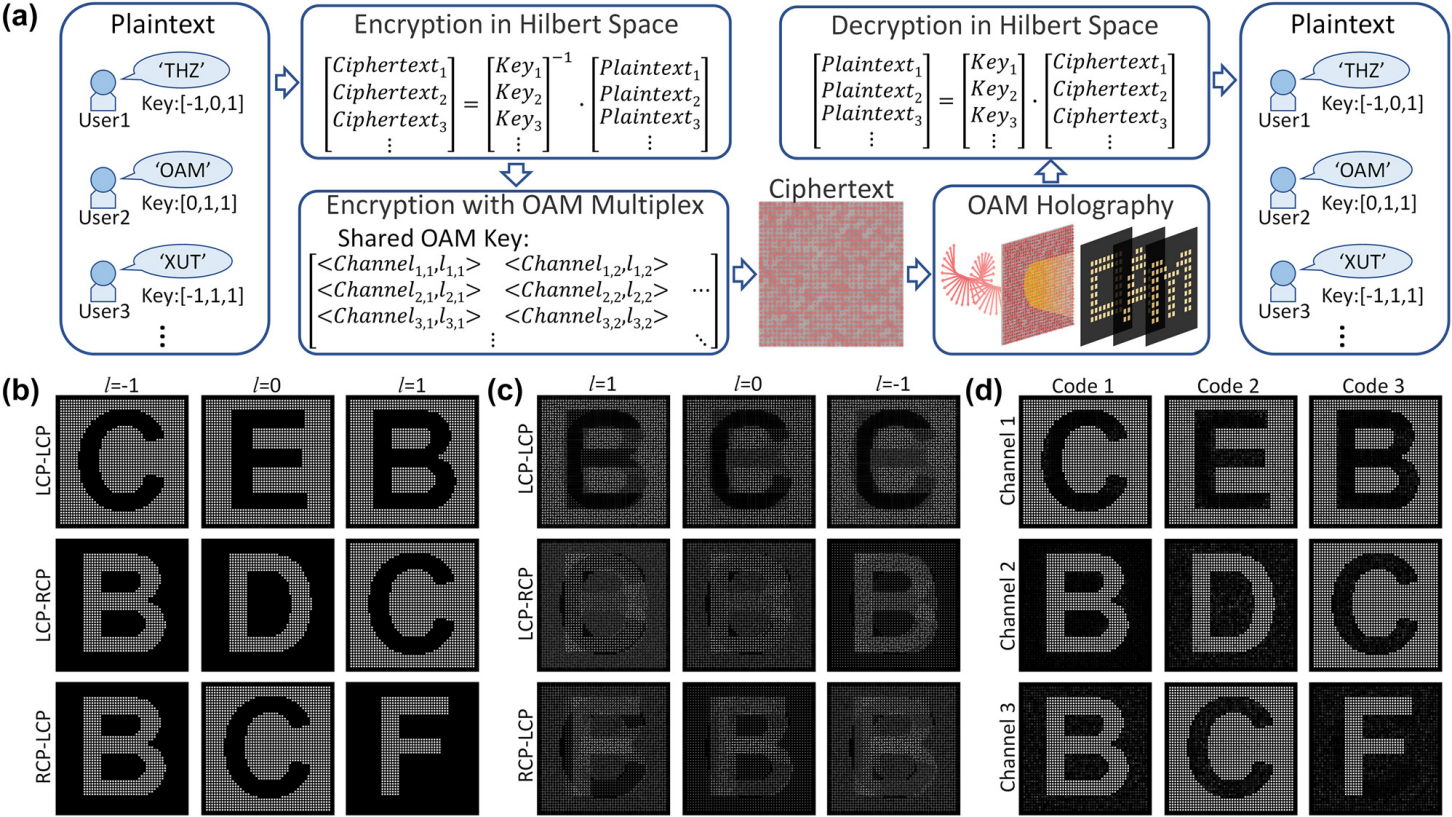
Schematic illustration of optical nested encryption based on proposed metasurface. (a) Process description of the optical nested encryption scheme. The plaintext is first converted into digital code, and then undergoes nested encryption through linear transformation in Hilbert space and OAM multiplexing, ultimately obtaining a metasurface that carries the ciphertext. The ciphertext can be decrypted using shared OAM keys and independent vector keys. (b) Holographic image corresponding to OAM Key. (c) Numerical reconstruction of holographic images from metasurface under different detection conditions. (d) Holographic images obtained through OAM selective mask sampling.

Our proposed OAM multiplexing holographic strategy based on multi-polarized channel metasurface can make full use of the degree of freedom of light, further increase the number of independent holographic image channels that can be realized by a single metasurface, which providing a platform to achieve high-capacity, high-security optical encryption. Different from optical encryption based only on DoFs, our proposed optical encryption strategy can freely choose the way to construct OAM holograms in different polarization channels, and can make holographic images reconstructed on different planes in space, which further improves the security of encrypted data. As a proof of concept, we propose an optically nested encryption scheme for high-capacity information transfer with extremely high security. Figure 7(a) shows the schematic diagram of the proposed optical nested encryption scheme, in which linear transformation and OAM multiplexing are used to realize the encryption and decryption of information. More specifically, in the encryption process, the plaintext of each user is first linearly transformed in the Hilbert space to obtain the ciphertext code. Then, according to the channels and topological charges in the shared OAM key, the holograms corresponding to the ciphertext codes are combined into OAM multiplexing holograms in multiple polarization channels, and finally a metasurface containing all information, termed OAM ciphertext, is obtained. The decryption process is the inverse of the encryption process. For each user, firstly retrieve a series of OAM holograms from OAM ciphertext according to the shared OAM key, and convert them into ciphertext codes, and then transform the ciphertext codes in Hilbert space with their own key. In this way, the user can obtain the plaintext carried in the nest encrypted OAM cipher. Given that the key of each user is only a part of the complete transformation matrix; each user can only obtain the projection of the corresponding dimension of the plaintext in the Hilbert space, which ensures that leaks will not occur between users using the same shared OAM key. Although each plaintext is converted to ciphertext code together in the encryption process, they actually use orthogonal channels defined by each user’s independent key in the Hilbert space. On this basis, multiple user groups can use different shared OAM keys to further improve the security of optical encryption.

As shown in Figure 7(b), there are 9 holographic images corresponding to the ciphertext code obtained by encrypting the plaintext (“THZ”, “XUT” – the abbreviation of Xi’an University of Technology, “OAM”) that needs to be sent to three users in Figure 6(a). These holographic images correspond to the channels and topological charges in the shared OAM key respectively. Numerical reconstruction of holographic images from OAM ciphertext under different detection conditions as shown in Figure 7(c). After resampling the holographic image in Figure 7(c) using the OAM selective mask (similar to the 2D Dirac array shown in Figure 3(c)), the holographic image corresponding to the shared OAM key is shown in Figure 7(d). As illustrated in Figure 7(d), the image closely mirrors the holographic representation corresponding to the ciphertext depicted in Figure 7(b). While the intensity of certain pixels exhibits minor variations, a pronounced contrast remains between the pixels, ensuring the encapsulated information remains distinctly perceptible. The letters in the holographic image correspond to the ciphertext code in the Hilbert space, where the letters “a” ∼ “z” of the positive film correspond to the numbers 1–26, and the letters “a” ∼ “z” of the negative film correspond to the numbers −1 ∼ −26. The inner product of each user’s key and ciphertext code in Hilbert space represents the corresponding plaintext, and the numbers 1–8 correspond to the string “AHMOTUXZ”. In order to improve the readability of the picture, we enlarge the pixel size of each sampling point in Figure 7(b) and (d).

## 3 Conclusions

In summary, our work proposes an OAM multiplexed holographic design based on a multi-channel complex amplitude modulation metasurface. By making full use of the SAM and OAM of photons, multiple polarization multiplexing channels combining with OAM multiplexing holography are constructed, and a variety of different types of OAM multiplexing holograms are constructed to verify its versatility. We propose an optical nested encryption scheme protected by SAM and OAM, which realizes secure parallel information transmission among multiple users. Our work provides a new design idea for high-capacity OAM multiplexing and multi-channel information encryption.
